# Effects of Longer-Term Mixed Nut Consumption on Lipoprotein Particle Concentrations in Older Adults with Overweight or Obesity

**DOI:** 10.3390/nu17010008

**Published:** 2024-12-24

**Authors:** Kevin M. R. Nijssen, Marco A. Chavez-Alfaro, Peter J. Joris, Jogchum Plat, Ronald P. Mensink

**Affiliations:** Department of Nutrition and Movement Sciences, NUTRIM Institute of Nutrition and Translational Research in Metabolism, Maastricht University Medical Center, 6229 ER Maastricht, The Netherlands; m.chavezalfaro@maastrichtuniversity.nl (M.A.C.-A.); p.joris@maastrichtuniversity.nl (P.J.J.); j.plat@maastrichtuniversity.nl (J.P.); r.mensink@maastrichtuniversity.nl (R.P.M.)

**Keywords:** apolipoproteins, cardiovascular disease, cholesterol, lipids, lipoprotein particles, mixed nuts, nuclear magnetic resonance spectrometry, triacylglycerol

## Abstract

Background: Recently, we reported that longer-term mixed nut intake significantly reduced serum total and low-density lipoprotein (LDL)-cholesterol, but these markers may not fully capture lipoprotein-related cardiovascular disease (CVD) risk. Objectives: This randomized, controlled, single-blinded, crossover trial in older adults with overweight or obesity examined the effects of longer-term mixed nut consumption on lipoprotein particle size, number, and lipid distribution. Methods: Twenty-eight participants (aged 65 ± 3 years; BMI 27.9 ± 2.3 kg/m^2^) completed two 16-week periods (control [no nuts] vs. mixed nuts (60 g/day: 15 g of walnuts, pistachios, cashews, and hazelnuts), separated by an 8-week washout. Plasma lipoprotein particle numbers, sizes, and lipid distributions across subclasses were analyzed using high-throughput nuclear magnetic resonance (NMR) spectroscopy. Results: Mixed nut consumption significantly reduced Apolipoprotein B (ApoB) concentrations (−0.07 g/L; *p* = 0.009), total cholesterol (−0.27 mmol/L; *p* = 0.047), non-HDL cholesterol (−0.28 mmol/L; *p* = 0.022), and total triacylglycerol (TAG) (−0.27 mmol/L; *p* = 0.008). Total very large-density lipoprotein (VLDL) particle numbers decreased by 24 nmol/L (*p* < 0.001), with reductions observed across all VLDL subclasses. Total LDL particle numbers (*p* = 0.044), specifically intermediate-density lipoprotein (IDL) (*p* = 0.002) and large LDL particles (*p* = 0.015), were also reduced, while HDL particle numbers and sizes were unaffected. The mixed nut intervention significantly reduced cholesterol concentrations across all VLDL subclasses and IDL (all *p* < 0.01), with no changes in LDL or HDL subclasses. TAG concentrations showed reductions across all lipoprotein subclasses (all *p* < 0.05). Conclusions: Longer-term mixed nut consumption may lower CVD risk in older adults and favorable shifts in apoB-containing lipoprotein subclasses towards a less atherogenic profile.

## 1. Introduction

Cardiovascular diseases (CVDs) remain the leading cause of morbidity and mortality worldwide, with atherosclerosis—the progressive accumulation of lipid-rich plaques within arterial walls—as a key underlying pathology. Hyperlipidemia, characterized by elevated concentrations of total cholesterol or low-density lipoprotein cholesterol (LDL-C) and triacylglycerol (TAG), is a major driver of atherosclerosis [1]. Traditionally, CVD risk has been evaluated using these circulating lipid markers. However, these markers may not fully capture the complexity of lipoprotein-related CVD risk [2]. Attention has also been paid to high-density lipoproteins cholesterol (HDL-C), but the role of HDL metabolism as related to CVD remains unclear [3]. Emerging evidence highlights the role of apolipoprotein B (ApoB)-containing lipoprotein particles, specifically very low-density lipoprotein (VLDL), intermediate-density lipoprotein (IDL), and LDL subclasses, as contributors to atherogenesis [4]. While LDL particles are recognized as pro-atherogenic [5], recent studies suggest that TAG- and cholesterol-rich particles such as VLDL and IDL, which significantly contribute to remnant cholesterol concentrations, are also strongly associated with CVD risk [6,7,8]. In this context, nuclear magnetic resonance (NMR)-based approaches offer more detailed profiling of these lipoprotein particles, yielding additional insights into CVD risk prediction beyond traditional lipid measurements [9,10,11].

Meta-analyses have demonstrated that regular nut consumption can improve cardiovascular outcomes by favorably affecting lipid profiles, including reductions in serum total cholesterol, LDL-C, and TAG concentrations [12,13,14]. The cardioprotective effects of nuts are attributed to their high content of unsaturated fats, fiber, and phytosterols, all of which are thought to beneficially influence lipid metabolism [15]. However, the number of dietary intervention studies investigating the effects of nuts on plasma lipoprotein subparticle distribution and concentration is limited. Previous large cohort studies, including the PREDIMED and WAHA trials, have suggested that higher nut intake is associated with reduced levels of atherogenic lipoproteins, particularly large VLDL and total LDL particles [16,17]. Nonetheless, these findings require further validation through randomized controlled trials (RCTs) in other study populations and with other nut types, which are essential for establishing causality in the relationship between nut consumption and changes in lipoprotein profiles. In our recent randomized, controlled, single-blinded, crossover trial involving older adults with overweight or obesity, we reported that long-term mixed nut intake (60 g/day of walnuts, pistachios, cashews, and hazelnuts) significantly reduced serum total and LDL-C concentrations using traditional lipid assessments [18]. Here, we extend these findings by examining the effects of this intervention on lipoprotein particle size, number, and lipid distribution across subclasses.

## 2. Materials and Methods

Approval for the study was obtained by the Medical Ethics Committee of University Hospital Maastricht and Maastricht University, which was registered on ClinicalTrials.gov (NCT04210869). The trial adhered to the Declaration of Helsinki guidelines and written informed consent was obtained from all participants. The study was conducted from January 2020 to December 2022.

### 2.1. Study Population

Details of the study have been published before [18,19]. Briefly, healthy men and postmenopausal women aged 60–70 years with a body mass index (BMI) of 25–35 kg/m^2^, classified as overweight or obese based on World Health Organization (WHO) criteria, were recruited via online advertisements, printed flyers, and databases of individuals who had participated in previous studies. Main eligibility criteria included stable body weight (<3 kg change in the past three months), office blood pressure < 160/100 mmHg, fasting plasma glucose < 7.0 mmol/L, total cholesterol < 8.0 mmol/L, and TAG < 4.5 mmol/L, and no blood donation eight weeks prior to screening and during the trial. Participants were excluded for nut allergies or intolerances, severe medical conditions (e.g., type 2 diabetes, cardiovascular disease), current or recent smoking (<1 year), use of medication or dietary supplements known to interfere with the study outcomes, alcohol or drug abuse, familial hypercholesterolemia, or participation in another biomedical study within one month prior to screening.

### 2.2. Study Design

The study had a randomized controlled, single-blinded, cross-over design. The intervention and control periods lasted 16 weeks and were separated by an 8-week washout period. Participants were randomly assigned to the intervention or control (no nuts) group in blocks of two or four stratified by sex. Throughout the intervention period, participants consumed daily sachets providing 60 g (359 kcal) of unsalted, unroasted mixed nuts (BasBoerNoten, Ridderkerk, the Netherlands). Each sachet contained 15 g walnuts, 15 g cashews, 15 g hazelnuts and 15 g pistachio (Table S1). Empty and unused sachets had to be returned during follow-up to assess compliance. Participants were instructed to avoid any products from a predefined list of foods with high amounts of n-3 PUFAs (e.g., other nuts, seeds, or fatty fish) and to follow the Dutch dietary guidelines [20]. Protocol deviations, health changes, medication use, and alcohol intake were recorded in participant diaries. Study visits occurred at baseline, as well as after 8 and 16 weeks, during which anthropometrics and fasting blood samples were collected via venipuncture. NaF plus Na_2_EDTA-containing tubes were immediately placed on ice and centrifuged (10 min at 1300× *g* at 4 °C). A validated food frequency questionnaire was filled out after 8 weeks and at follow-up, and results were averaged to assess energy and nutrient intakes based on the Dutch Food Composition Database, as described before [18]. After centrifugation, plasma samples were distributed in aliquots, snap-frozen, and stored at −80 °C until analysis. All measurements were performed in quiet, temperature-controlled rooms (20 °C) following 12-h fasting, and 48-h alcohol and severe exercise abstention. Participants had to come to the Metabolic Research Unit Maastricht (MRUM) by car or public transport.

### 2.3. Nuclear Magnetic Resonance Spectroscopy

Lipoprotein and metabolite profiling of plasma samples was performed using a high-throughput NMR metabolomics platform (Nightingale Health Ltd., Helsinki, Finland). All metabolites were measured in a single experimental setup that allowed for the simultaneous quantification of lipids and lipoproteins for the analyses: the concentrations of ApoB and apolipoprotein A1 (ApoA1); of lipoprotein concentrations (including VLDL, IDL, LDL, and HDL) and 13 subclasses of these lipoproteins, as defined by particle size; the average lipoprotein diameter; and the total lipid concentrations (including cholesteryl esters, free cholesterol, phospholipids, and TAG) of each lipoprotein subclass. This platform has been extensively used for metabolic profiling in epidemiological studies related to CVD risk [10,11,21,22,23]. Therefore, we limited our analysis to the biomarkers reported by this platform.

### 2.4. Statistical Analysis

Data were presented as means ± standard deviations (SDs) unless otherwise indicated. At least 27 participants were required to detect a 7.5% change in fasting cerebral blood flow, the primary outcome of the study [19], which was also sufficient for detecting changes in total cholesterol and LDL-C concentrations based on previous data [18]. Linear mixed models were used, including treatment, time, period, and sex as fixed factors, with participants as a random factor and baseline values as covariates. Time*treatment interactions provided information if the treatment effect was comparable at all timepoints. However, the interaction was omitted from all statistical models as it never reached statistical significance. Residual covariance structures were selected based on maximum likelihood estimation using Akaike’s Information Criteria. Carry-over effects were examined by including treatment order as a fixed factor, but no significant effects were found, and treatment order was therefore omitted from all models. Pearson’s correlations were used to compare the NMR-based results with our previous colorimetric lipid and lipoprotein assays [18]. All analyses were completed using SPSS (IBM Corp., IBM SPSS Statistics, V26, Armonk, NY, USA), with *p*-values ≤ 0.05 were considered statistically significant.

## 3. Results

### 3.1. Study Participants

The CONSORT flow diagram is shown in Figure S1. A total of 28 participants (14 men and 14 women, aged 65 ± 3 years; BMI: 27.9 ± 2.3 kg/m^2^) completed the study. Baseline clinical and biochemical characteristics have been reported previously [19]. There were no significant differences in body weight (0.3 kg; 95% CI: −0.4 to 1.0; *p* = 0.405) or BMI (0.2 kg/m^2^; 95% CI: −0.1 to 0.4; *p* = 0.251) between treatments, and no serious adverse events or protocol deviations were reported. Compliance, as assessed by returned sachets, was excellent with a median compliance rate of 98% (IQR: 93–100%). Total energy and nutrient intakes during both the mixed nut and control periods were comparable, with no significant differences in total energy or protein intake (Table S2), as previously reported [18]. However, compared with the control, mixed nut consumption reduced carbohydrate intake by 4.9 En% (95% CI: −7.1 to −2.5; *p* < 0.001). Total fat intake was 5.6 En% higher (95% CI: 3.2 to 8.1; *p* < 0.001), with lower intake of saturated fatty acids and higher intakes of cis-monounsaturated fatty acids, cis-polyunsaturated fatty acids, linoleic acid and alpha-linolenic acid (all *p* < 0.01).

### 3.2. Comparison Between NMR Results with Colorimetric Assays

**Figure 1:**
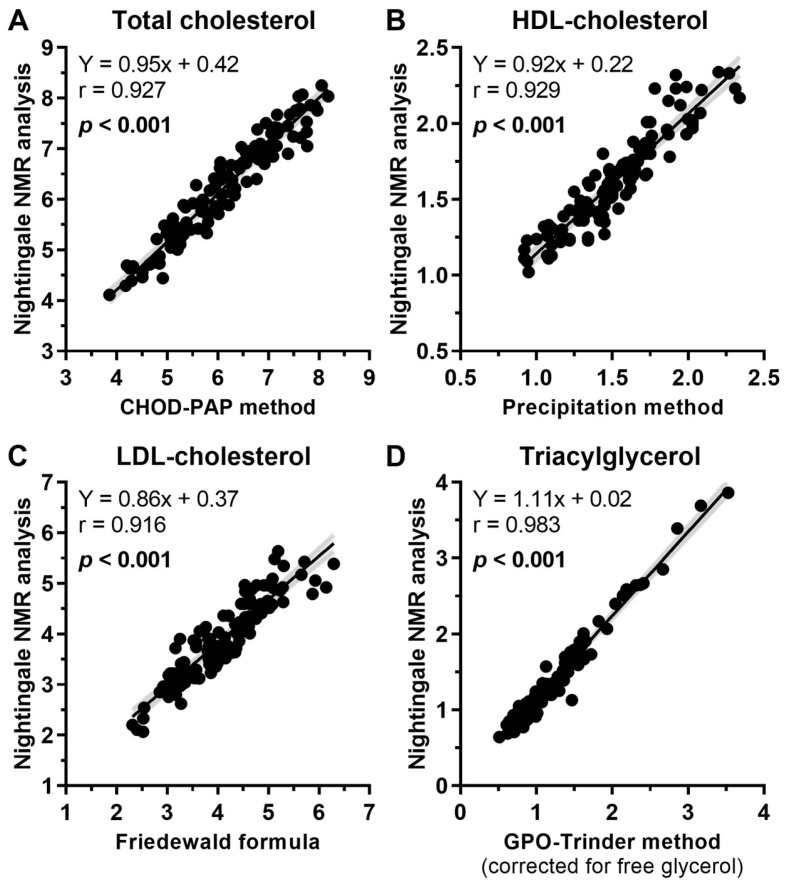
Comparison between Nuclear Magnetic Resonance (NMR) results with colorimetric assays. These latter results were previously reported [18]. High positive correlations were found for (**A**) total cholesterol (r = 0.927, *p* < 0.001), (**B**) HDL-C (r = 0.929, *p* < 0.001), (**C**) LDL-C (r = 0.916, *p* < 0.001), and (**D**) total triacylglycerol (r = 0.983, *p* < 0.001).

Total cholesterol, HDL cholesterol, and total triacylglycerol concentrations were analyzed by NMR and colorimetrically, while LDL-C was also calculated with the Friedewald formula [24] using the data from the colorimetric assays. These calorimetric results have already been reported [18]. High positive correlations were found for total cholesterol (r = 0.927, *p* < 0.001), HDL-C (r = 0.929, *p* < 0.001), LDL-C (r = 0.916, *p* < 0.001), and total triacylglycerol (r = 0.983, *p* < 0.001) between the NMR and colorimetric assays (Figure 1A–D).

### 3.3. Total Plasma Lipids and (Apo)lipoproteins

Table 1 presents the concentrations of total plasma lipids and (apo)lipoproteins. Compared to the control period, the mixed nut intervention decreased concentrations of total cholesterol (−0.27 mmol/L; 95% CI: −0.53 to 0.00; *p* = 0.047), total non-HDL cholesterol (−0.28 mmol/L; 95% CI: −0.52 to −0.04; *p* = 0.022), total TAG (−0.27 mmol/L; 95% CI: −0.47 to −0.07; *p* = 0.008), total phospholipids (−0.19 mmol/L; 95% CI: −0.32 to −0.06; *p* = 0.005), and total free cholesterol (−0.10 mmol/L; 95% CI: −0.17 to −0.02; *p* = 0.001). Plasma apoB concentrations (−0.07 g/L; 95% CI: −0.12 to −0.02; *p* = 0.009) and the apoB/apoA1 ratio (−0.05; 95% CI: −0.08 to −0.01; *p* = 0.009) were also significantly reduced. No statistically significant differences were observed for total LDL-C, total HDL-C, cholesteryl esters, or apoA1.

### 3.4. Lipoprotein Particle Numbers and Sizes

**Figure 2:**
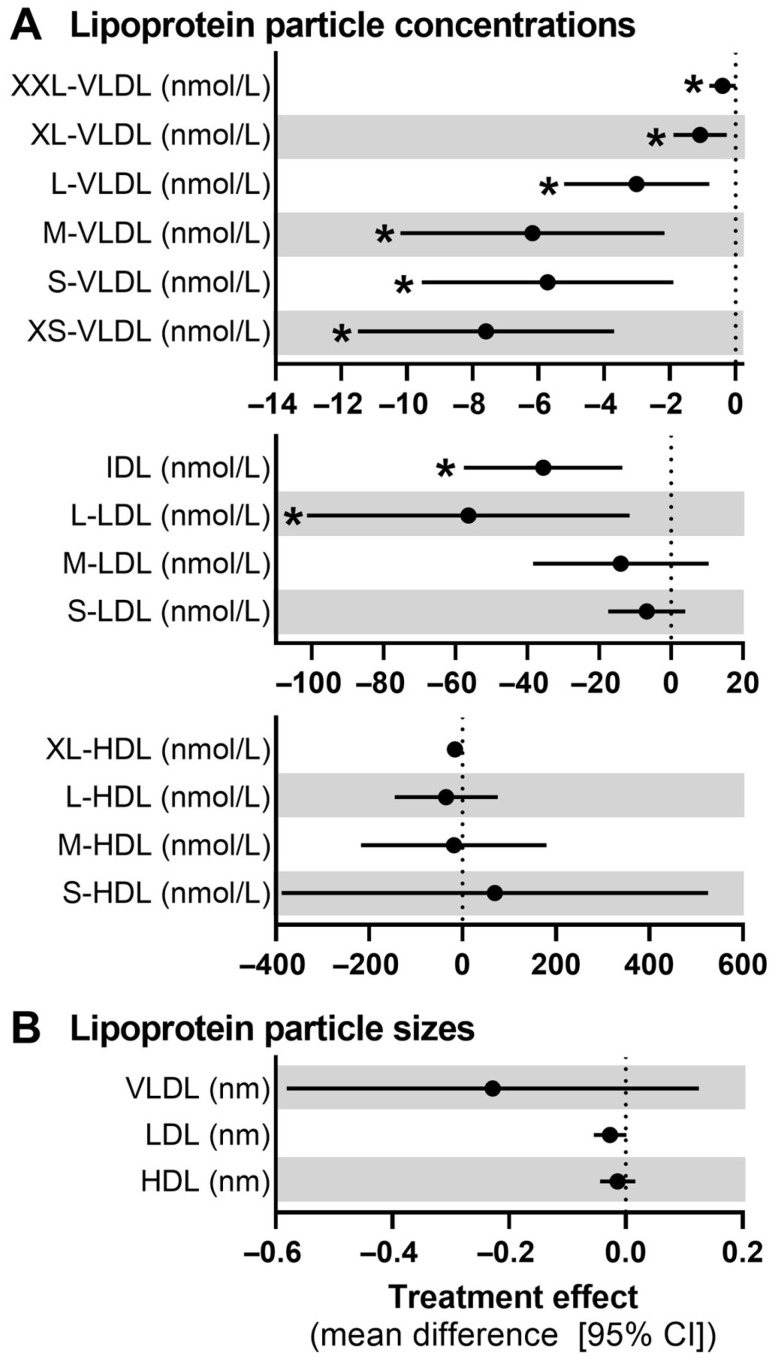
Mean difference (95% CI) in (**A**) lipoprotein particle concentrations in VLDL, IDL, LDL, and HDL subclasses and (**B**) lipoprotein particle sizes for VLDL, LDL, and HDL after the mixed nut intervention and control period (*n* = 28). An asterisk (*) denoted significant treatment effects (*p* < 0.05) based on linear mixed-model analysis with random intercepts, period, sex, time, and treatment as fixed factors, participant as random factor, and baseline values as covariates.

The NMR analysis revealed significant reductions in total VLDL particle numbers following the mixed nut intervention, with a decrease of 24 nmol/L (95% CI: −38 to −11, *p* < 0.001) (Table S3). This reduction was observed across all VLDL subclasses (Figure 2A, all *p* < 0.05). Similarly, total LDL particle numbers decreased by 77 nmol/L (95% CI: −152 to −2, *p* = 0.044), driven by significant reductions in IDL particles (−35 nmol/L; 95% CI: −58 to −13, *p* = 0.002) and L-LDL particles (−56 nmol/L; 95% CI: −101 to −12, *p* = 0.015), but no significant changes were found in M-LDL or S-LDL particle numbers. Total HDL particle numbers were not significantly different between the intervention and control periods. Additionally, no significant changes were observed in the mean particle sizes of VLDL, LDL, or HDL (Figure 2B).

### 3.5. Lipoprotein Concentrations

**Figure 3:**
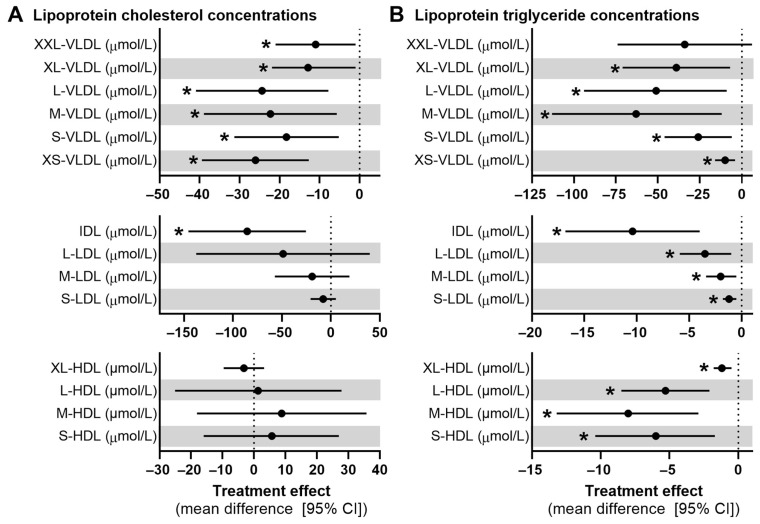
Mean difference (95% CI) in (**A**) lipoprotein cholesterol concentrations and (**B**) lipoprotein triacylglyceride concentrations in VLDL, IDL, LDL, and HDL subclasses after the mixed nut intervention and control period (*n* = 28). An asterisk (*) denoted significant treatment effects (*p* < 0.05) based on linear mixed-model analysis with random intercepts, period, sex, time, and treatment as fixed factors, participant as random factor, and baseline values as covariates.

The concentrations of cholesterol and TAG within VLDL, IDL, LDL, and HDL subclasses are summarized in Figure 3 and Table S4. The mixed nut intervention led to significant reductions in cholesterol concentrations across all VLDL subclasses and IDL, while no significant effects were noted for cholesterol concentrations in LDL or HDL subclasses (Figure 3A). Specifically, XL-VLDL cholesterol decreased by 13 µmol/L (95% CI: −10 to −6; *p* = 0.006), L-VLDL by 24 µmol/L (95% CI: −41 to −8; *p* = 0.005), M-VLDL by 22 µmol/L (95% CI: −39 to −6; *p* = 0.009), S-VLDL by 18 µmol/L (95% CI: −31 to −5; *p* = 0.007), XS-VLDL by 26 µmol/L (95% CI: −39 to −13; *p* < 0.001), and IDL by 86 µmol/L (95% CI: −146 to −26; *p* = 0.006). Similar reductions were observed in TAG concentrations across all lipoprotein subclasses (Figure 3B, all *p* < 0.05). Decreases were also observed for phospholipids, free cholesterol, and cholesteryl esters, specifically in XL-VLDL, L-VLDL, S-VLDL, XS-VLDL, and IDL subclasses (all *p* < 0.01) (Table S4). Relative lipid concentrations were not significantly different across VLDL, IDL, or LDL subclasses. However, for most HDL subclasses there were relative increases in cholesteryl esters and reductions in TAG (Table S5).

## 4. Discussion

In this randomized, controlled, crossover trial involving older adults with overweight and obesity, we explored the long-term effects of mixed nut consumption on lipoprotein particle numbers, sizes, and lipid content across various lipoprotein subclasses using an advanced NMR metabolomics platform. Our results indicated significant improvements in serum lipid profiles, with reductions in plasma ApoB, total cholesterol, non-HDL cholesterol, and total TAG. Moreover, we observed a decline in the number of total VLDL, IDL, and LDL particles, as well as decreases in cholesterol and TAG content within different lipoprotein subclasses. These findings extend our previous analyses by providing more detailed insights into the serum lipid-modulating effects of nuts in the prevention of CVD [18].

We observed a significant reduction in ApoB concentrations, due to decreases in the number of VLDL, IDL, and LDL particles, each of which contains one molecule of ApoB. This reduction in ApoB implies a decrease in the number of atherogenic lipoprotein particles, as ApoB concentrations are strongly associated with CVD risk [25]. Since mean particle sizes of VLDL and LDL remained unchanged, it suggests that the relative reductions were distributed evenly across the various VLDL and LDL subclasses. Indeed, this was observed, although reductions in M-LDL and S-LDL particle numbers did not reach statistical significance. Our findings on ApoB align closely with those recently reported by Nishi et al. [14], which concluded that a median daily dose of 45.4 g/d of tree nuts reduced ApoB by 0.04 g/L. In our study, we observed a decrease of 0.07 g/L with a daily intake of 60 g of mixed nuts. Consistent with previous meta-analyses [13,14], we observed no effect on ApoA1 concentrations.

Our findings on the reduction in lipoprotein particle numbers largely align with prior observational and intervention studies on nut consumption [12]. For instance, an observational study within the PREDIMED trial found that increasing nut intake (~14–30 g/day) over a one-year period was associated with reduced numbers of very large VLDL and total LDL particle numbers compared to low nut intake [16]. Similarly, the WAHA trial, the largest nut intervention to date, reported that consuming 30 to 60 g/day of walnuts over two years decreased total LDL particle concentrations, although primarily impacting small LDL particles, along with reductions in total cholesterol, IDL, and LDL-C [17]. Other intervention studies further support these findings, showing that mixed nut consumption including walnuts lowered total LDL particle numbers [26], while pistachios reduced both VLDL and LDL particle numbers in another trial [27]. Though large VLDL particles have for long been recognized as the most pro-atherogenic [28], recent research indicates that smaller VLDL and IDL particles, which substantially contribute to remnant cholesterol concentrations, may be among the most atherogenic TAG-rich lipoproteins [6,7]. In fact, remnant lipoproteins have been associated with CVD, independently of LDL particles or LDL-C levels [7]. Additionally, the number of LDL particles has been suggested to be a stronger predictor of CVD risk than LDL-C in prospective studies [29]. Altogether, our results indicate that regular nut consumption may favorably alter lipoprotein subclass profiles, thereby reducing lipoprotein-related CVD risk.

Based on NMR analysis, we observed a significant decrease in plasma TAG concentrations by 0.27 mmol/L following mixed nut consumption, with reductions observed in each VLDL, LDL, and HDL subclass. This contrasts with our previously reported colorimetric analyses, where the decrease of 0.15 mmol/L in TAG concentrations did not reach statistical significance [18]. The reason for this apparent discrepancy is unclear, especially given the strong correlation between the NMR and colorimetric assays. However, the magnitude of the reduction for both methods also aligns with average decreases reported in meta-analyses on nut consumption and TAG concentrations [12,13,14]. Interestingly, earlier, we reported a 15% reduction in intrahepatic lipid content within this trial [18]. A decrease in intrahepatic lipids may reduce hepatic secretion of VLDL particles, thereby contributing to the decrease in TAG concentrations and a lower CVD risk profile [30].

Comparable to our previous colorimetric analyses [18], the NMR data showed that mixed nut consumption significantly reduced total cholesterol concentrations by 0.27 mmol/L. This corroborates with already plenty of evidence that regular tree nut consumption reduces total cholesterol [13,14,31]. Interestingly, a statistically significant cholesterol reduction was present within each VLDL subclass, as well as within the IDL and large LDL lipoproteins. For the NMR analyses, effects on LDL-C did not reach significance, a finding we reported before using the Friedewald formula [18,24]. It must be noted that the Friedewald formula defines LDL-C on the basis of density, and may include, to varying degrees, IDL and small VLDL particles [32], whereas in the NMR analyses, LDL concentrations are defined on particle sizes [4], which may to some extent explain the discrepancy between the results.

HDL functionality may play a more significant role in reducing CVD risk than simply increasing HDL-C or ApoA1 concentrations [33,34]. This functionality refers to multiple metabolic pathways including the reverse cholesterol transport pathway, which relates to HDL’s capacity to promote cholesterol efflux. It is influenced by particle characteristics, such as size and phospholipid composition [35]. In our study, we observed no changes in total HDL particle numbers or sizes, and the composition of the different phospholipids was not assessed. Furthermore, a change in HDL lipid composition, such as TAG enrichment and phospholipid reduction, can impair cholesterol efflux capacity [36]. We found reduced TAG content across all HDL subclasses, suggesting a possible positive effect on HDL functionality. In addition, shifts in the relative lipid-to-total lipid concentrations within these subclasses suggest that TAG may have been exchanged for cholesteryl esters. Nevertheless, HDL functionality was not directly measured in this study, highlighting a key area for future research.

The strengths and limitations of this study have been discussed before [18,19]. While this study did not directly assess hard clinical (CVD) outcomes, a key strength was the well-controlled study design and use of advanced NMR metabolomics, enabling detailed profiling of validated predictors of CVD risk and atherogenesis [3,4,5,6,7,8], thereby offering insights into the lipid-modulating effects of mixed nuts. Furthermore, the study did not observe significant differences in triacylglycerol concentrations when using colorimetric methods compared to NMR, and the reason for this discrepancy remains unclear. Importantly, the intervention did not significantly alter body weight or total energy intake, suggesting that participants compensated for the additional calories from nuts, possibly due to their high satiety value [37]. Moreover, the findings are specific to a well-defined population of older adults with overweight or obesity who are at increased cardiovascular risk but not yet diagnosed or treated for chronic or metabolic diseases. Therefore, our findings build upon previous evidence that nut consumption is associated with favorable lipid profiles in the primary prevention of CVD risk before the onset of the disease. However, further research is necessary to determine whether these findings are generalizable to other populations; for example, those using lipid-lowering medication. Lastly, our study was not statistically powered to conduct subgroup analyses, such as exploring potential sex differences, highlighting the need for future research with larger sample sizes.

## 5. Conclusions

In conclusion, our study in older adults with overweight or obesity indicates that long-term mixed nut consumption may lower CVD risk through a favorable shift in apoB-containing lipoprotein subclass numbers towards a less atherogenic profile, measured using an NMR metabolomic platform. An unexpected discrepancy was present between NMR and colorimetrically determined TAG concentrations. Future studies should further explore whether regular nut intake impacts HDL functionality to better define its role in CVD prevention strategies.

## Figures and Tables

**Table 1:** Total plasma lipids and apolipoproteins following the mixed nut intervention and control period in older adults ^1^.

	Mixed Nut Intervention			Control Intervention			Treatment Effect ^2^
	Week 0	Week 8	Week 16	Week 0	Week 8	Week 16	
Total lipid concentrations							
Total lipids (mmol/L)	11.8 ± 1.8	10.7 ± 1.9	10.7 ± 1.9	11.3 ± 1.8	11.0 ± 1.8	11.2 ± 2.0	−0.8 [−1.3, −0.2], *p* = 0.005
Total cholesterol (mmol/L)	6.26 ± 0.98	5.97 ± 0.96	6.02 ± 1.07	6.33 ± 1.02	6.17 ± 0.97	6.28 ± 1.06	−0.27 [−0.53, 0.00], *p* = 0.047
Cholesteryl esters (mmol/L)	4.57 ± 0.70	4.39 ± 0.68	4.42 ± 0.77	4.64 ± 0.74	4.51 ± 0.69	4.59 ± 0.76	−0.17 [−0.36, 0.02], *p* = 0.081
Free cholesterol (mmol/L)	1.69 ± 0.28	1.59 ± 0.28	1.60 ± 0.30	1.69 ± 0.29	1.65 ± 0.28	1.68 ± 0.31	−0.10 [−0.17, −0.02], *p* = 0.011
Total non-HDL-C (mmol/L)	4.65 ± 0.92	4.36 ± 0.86	4.40 ± 0.93	4.69 ± 0.92	4.56 ± 0.90	4.66 ± 0.96	−0.28 [−0.52, −0.04], *p* = 0.022
Total VLDL-C (mmol/L)	0.89 ± 0.25	0.77 ± 0.22	0.78 ± 0.21	0.85 ± 0.22	0.83 ± 0.23	0.86 ± 0.25	−0.12 [−0.19, −0.05], *p* = 0.001
Total LDL-C (mmol/L)	3.86 ± 0.80	3.66 ± 0.70	3.67 ± 0.80	3.95 ± 0.81	3.84 ± 0.77	3.95 ± 0.83	−0.16 [−0.36, 0.05], *p* = 0.129
Total HDL-C (mmol/L)	1.61 ± 0.35	1.62 ± 0.30	1.62 ± 0.34	1.64 ± 0.31	1.60 ± 0.29	1.62 ± 0.33	0.01 [−0.05, 0.08], *p* = 0.645
Total TAG (mmol/L)	1.50 ± 0.67	1.25 ± 0.57	1.23 ± 0.38	1.35 ± 0.49	1.35 ± 0.63	1.39 ± 0.64	−0.27 [−0.47, −0.07], *p* = 0.008
Total phospholipids (mmol/L)	3.62 ± 0.49	3.45 ± 0.54	3.45 ± 0.52	3.59 ± 0.50	3.53 ± 0.49	3.58 ± 0.54	−0.19 [−0.32, −0.06], *p* = 0.005
Apolipoproteins							
ApoB (g/L)	1.12 ± 0.22	1.05 ± 0.20	1.06 ± 0.22	1.12 ± 0.21	1.10 ± 0.22	1.12 ± 0.22	−0.07 [−0.12, −0.02], *p* = 0.009
ApoA1 (g/L)	1.68 ± 0.26	1.67 ± 0.25	1.66 ± 0.26	1.69 ± 0.24	1.66 ± 0.24	1.68 ± 0.26	−0.01 [−0.06, 0.04], *p* = 0.623
ApoB to ApoA1 ratio	0.69 ± 0.19	0.64 ± 0.15	0.65 ± 0.15	0.67 ± 0.16	0.68 ± 0.18	0.69 ± 0.18	−0.05 [−0.08, −0.01], *p* = 0.008

^1^ Values are means ± SDs; *n* = 28. HDL-C, high-density lipoprotein cholesterol; VLDL-C, very low-density lipoprotein cholesterol; LDL-C, low-density lipoprotein cholesterol; TAG, triacylglycerol; ApoB, apolipoprotein B; ApoA1, apolipoprotein A1. ^2^ Linear mixed-model analysis with random intercepts. Period, sex, time, treatment, and time × treatment interaction were used as fixed factors, participant as a random factor, and baseline values as covariates. The interaction term was not statistically significant and, therefore, omitted from the final model. *p* values for the effect of treatment (mean difference [95% CI] between the mixed nut and control interventions) were reported.

## Data Availability

The datasets used and/or analyzed during the current study are available from the corresponding author upon reasonable request due to legal reasons.

## Supplementary Materials

The following supporting information can be downloaded at: https://www.mdpi.com/article/10.3390/nu17010008/s1, Figure S1: CONSORT flow diagram; Table S1: Nutritional composition of mixed nuts (BasBoerNoten; Ridderkerk, the Netherlands); Table S2: Daily energy and nutrient intake following the mixed nut intervention and control period in older adults; Table S3: Total lipoprotein particle numbers and sizes following the mixed nut intervention and control period in older adults; Table S4: Total lipoprotein subclass concentrations following the mixed nut intervention and control period in older adults; Table S5: Relative lipid concentrations within lipoprotein subclasses following the mixed nut intervention and control period in older adults.
