# Supplementary material for: Effects of Longer-Term Mixed Nut Consumption on Lipoprotein Particle Concentrations in Older Adults with Overweight or Obesity
Source: Nutrients. 2024 Dec 24;17(1):8. doi: 10.3390/nu17010008 (PMC11723242; doi:10.3390/nu17010008)
Supplement: Supplementary file 1 [file nutrients-17-00008-s001.zip › nutrients-3353095-supplementary.pdf]

Table S1. Nutritional composition of mixed nuts (BasBoerNoten; Ridderkerk, the Netherlands)[1].

	**Per portion** (60 g)	**Walnut** (60 g)	**Cashew** (60 g)	**Hazelnut** (60 g)	**Pistachio** (60 g)
Total energy (Kcal)	359	98	83	94	84
Protein (g)	10.3	2.3	2.7	2.2	3.0
Carbohydrates (g)	13.2	2.1	4.5	2.5	4.1
Total fat (g)	32.3	9.8	6.6	9.1	6.8
Total SFA (g)	3.6	0.9	1.2	0.7	0.9
Total MUFA (g)	15.3	1.3	3.6	6.9	3.5
Total PUFA (g)	11.6	7.1	1.2	1.2	2.2
Total linoleic acid (g)	10.0	5.7	1.2	2.0	1.2
Total α-linolenic acid (g)	1.4	1.4	0.0	0.0	0.0
Fibers (g)	4.5	1.0	0.5	1.5	1.6

[1] SFA, saturated fatty acids; MUFA, monounsaturated fatty acids; PUFA, polyunsaturated fatty acids.

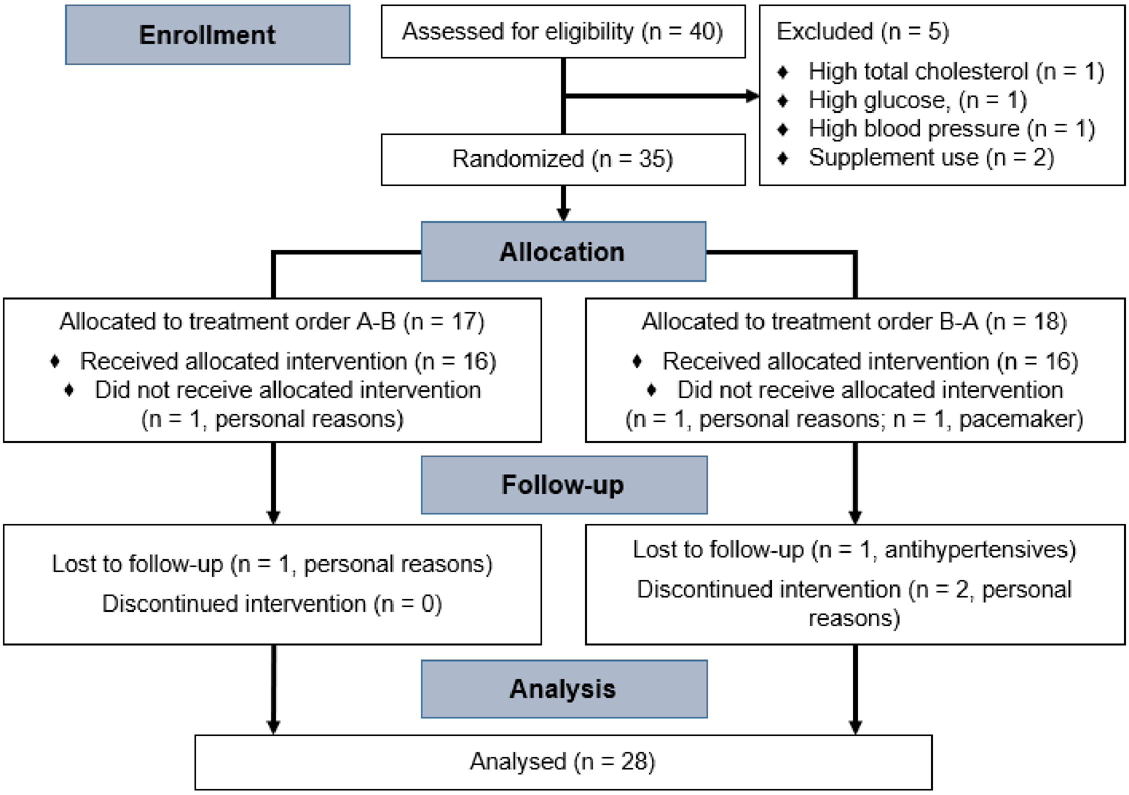

Figure S1. CONSORT flow diagram. In total, 35 subjects were eligible to participate who were randomized for treatment order (A = Mixed nuts intervention, B = Control intervention). During the intervention seven participants dropped out, resulting in a total of 28 subjects for the analysis. This is a figure adapted from our previous manuscript [18].

Table S2. Daily energy and nutrient intake following the mixed nut intervention and control period in older adults[1].

	Mixed nut intervention	Control intervention	Treatment effect[2]
Total energy (Kcal)	2277 ± 507	2301 ± 495	-25 [-297, 247], $p = 0.853$
Protein (En%)	16.5 ± 2.3	16.1 ± 2.1	0.5 [-0.7, 1.6], $p = 0.394$
Carbohydrates (En%)	37.2 ± 3.8	42.0 ± 5.2	-4.9 [-7.1, -2.5], **$p < 0.001$**
Total fat (En%)	42.1 ± 3.8	36.5 ± 5.6	5.6 [3.2, 8.1], **$p < 0.001$**
Total SFA (En%)	10.9 ± 3.0	12.8 ± 2.3	-1.8 [-3.2, -0.5], **$p = 0.010$**
Total cis-MUFA (En%)	16.5 ± 2.1	13.0 ± 2.8	3.4 [2.2, 4.7], **$p < 0.001$**
Total cis-PUFA (En%)	10.8 ± 1.8	7.3 ± 2.4	3.6 [2.5, 4.7], **$p < 0.001$**
Linoleic acid (En%)	9.2 ± 1.6	5.9 ± 1.9	3.3 [2.4, 4.2], **$p < 0.001$**
α-Linolenic acid (En%)	0.9 ± 0.3	0.7 ± 0.3	0.2 [0.1, 0.3], **$p < 0.001$**
Cholesterol (mg/MJ)	27.0 ± 7.8	29.8 ± 7.6	-2.7 [-6.6, 1.1], $p = 0.160$
Fibers (g)	27.4 ± 5.2	26.0 ± 5.7	1.4 [-1.4, 4.2], $p = 0.334$

[1] Values are means ± SDs; n = 28. The investigational product was included, and data after 8 weeks and follow up were averaged. En%, energy percentage. SFA, saturated fatty acids; MUFA, monounsaturated fatty acids; PUFA, polyunsaturated fatty acids.

[2] Linear mixed model analysis with random-intercept. Period, gender, and treatment were used as fixed factors, and participant as random factor. P-values for the effect of treatment (mean difference [95% CI] between the mixed nut and control intervention) were reported.

Table S3. Total lipoprotein particle numbers and sizes following the mixed nut intervention and control period in older adults[1].

	Mixed nut intervention			Control intervention			Treatment effect[2]
	Week 0	Week 8	Week 16	Week 0	Week 8	Week 16	
**Total lipoprotein particle concentrations**							
Total VLDL particles (nmol/L)	178 ± 49	155 ± 45	156 ± 39	168 ± 41	166 ± 48	170 ± 47	-24 [-38, -11], **p < 0.001**
XXL (nmol/L)	1.1 ± 1.4	0.7 ± 0.8	0.7 ± 0.7	0.8 ± 0.9	0.8 ± 1.0	0.9 ± 1.3	-0.4 [-0.8, 0.0], **p = 0.049**
XL (nmol/L)	4.1 ± 2.8	3.2 ± 2.1	3.1 ± 1.6	3.6 ± 2.0	3.6 ± 2.5	3.8 ± 2.8	-1.1 [-1.9, -0.3], **p = 0.010**
L (nmol/L)	13 ± 8	11 ± 6	11 ± 4	12 ± 6	12 ± 7	12 ± 8	-3 [-5, -1], **p = 0.009**
M (nmol/L)	48 ± 15	42 ± 14	43 ± 11	47 ± 13	46 ± 15	47 ± 15	-6 [-10, -2], **p = 0.003**
S (nmol/L)	47 ± 14	41 ± 13	41 ± 11	44 ± 11	43 ± 14	44 ± 12	-6 [-10, -2], **p = 0.004**
XS (nmol/L)	64 ± 15	57 ± 13	57 ± 14	60 ± 12	61 ± 14	62 ± 15	-8 [-12, -4], **p < 0.001**
Total LDL particles (μmol/L)	1607 ± 320	1527 ± 280	1550 ± 316	1632 ± 311	1595 ± 312	1624 ± 322	-77 [-152, -2], **p = 0.044**
IDL (μmol/L)	407 ± 84	371 ± 71	368 ± 74	393 ± 76	389 ± 73	397 ± 47	-35 [-57, -13], **p = 0.002**
L-LDL (μmol/L)	987 ± 188	937 ± 162	948 ± 188	997 ± 187	982 ± 170	999 ± 187	-56 [-101, -12], **p = 0.015**
M-LDL (μmol/L)	397 ± 95	375 ± 87	384 ± 89	406 ± 90	391 ± 105	399 ± 95	-14 [-38, 10], p = 0.256
S-LDL (μmol/L)	222 ± 46	214 ± 41	218 ± 45	228 ± 43	222 ± 48	226 ± 46	-7 [-17, 4], p = 0.211
Total HDL particles (μmol/L)	18439 ± 2789	18376 ± 2625	18339 ± 2657	18760 ± 2468	18226 ± 2532	18440 ± 2733	18 [-640, 676], p = 0.956
XL-HDL (μmol/L)	262 ± 92	248 ± 81	251 ± 88	254 ± 88	259 ± 78	260 ± 86	-16 [-33, 0], p = 0.053
L-HDL (μmol/L)	1717 ± 790	1648 ± 678	1695 ± 751	1700 ± 694	1689 ± 649	1704 ± 731	-35 [-145, 75], p = 0.524
M-HDL (μmol/L)	4648 ± 1034	4619 ± 961	4585 ± 978	4687 ± 910	4548 ± 953	4614 ± 1026	-19 [-218, 180], p = 0.850
S-HDL (μmol/L)	11812 ± 1607	11825 ± 1487	11807 ± 1380	12119 ± 1396	11730 ± 1489	11862 ± 1500	69 [-387, 525], p = 0.763
**Lipoprotein particle size**							
VLDL size (nm)	38.8 ± 1.2	38.6 ± 0.9	38.6 ± 0.8	38.8 ± 0.9	38.7 ± 1.1	38.7 ± 1.2	-0.2 [-0.6, 0.1], p = 0.201
LDL size (nm)	23.9 ± 0.1	23.9 ± 0.1	23.9 ± 0.1	23.9 ± 0.1	24.0 ± 0.1	23.9 ± 0.1	0.0 [-0.1, 0.0], p = 0.062
HDL size (nm)	9.6 ± 0.2	9.6 ± 0.2	9.6 ± 0.2	9.6 ± 0.2	9.6 ± 0.2	9.6 ± 0.2	0.0 [0.0, 0.0], p = 0.340

[1] Values are means ± SDs; n = 28. VLDL, very low-density lipoprotein; LDL, low-density lipoprotein; HDL, high-density lipoprotein.

[2] Linear mixed-model analysis with random intercepts. Period, sex, time, treatment, and time x treatment interaction were used as fixed factors, participant as random factor, and baseline values as covariates. The interaction term was not statistically significant and, therefore, omitted from the final model. P values for the effect of treatment (mean difference [95% CI] between the mixed nut and control interventions) were reported.

Table S4. Total lipoprotein subclass concentrations following the mixed nut intervention and control period in older adults[1].

	Mixed nut intervention			Control intervention			Treatment effect[2]
	Week 0	Week 8	Week 16	Week 0	Week 8	Week 16	
**XXL-VLDL subclass concentrations** (average particle diameter > 75.0 nm)							
Lipids (μmol/L)	141 ± 194	85 ± 109	86 ± 84	106 ± 113	97 ± 137	114 ± 178	-51 [-108, 5], p = 0.075
Cholesterol (μmol/L)	34 ± 36	22 ± 13	23 ± 18	26 ± 23	25 ± 26	28 ± 31	-11 [-21, -1], **p = 0.032**
Cholesteryl esters (μmol/L)	19 ± 20	12 ± 13	13 ± 10	15 ± 13	14 ± 15	16 ± 18	-6 [-12, -1], **p = 0.020**
Free cholesterol (μmol/L)	15 ± 16	9 ± 10	10 ± 8	11 ± 10	10 ± 11	12 ± 14	-4 [-9, 0], p = 0.060
Triacylglycerol (μmol/L)	88 ± 134	53 ± 72	52 ± 55	66 ± 75	61 ± 94	72 ± 125	-34 [-74, 6], p = 0.091
Phospholipids (μmol/L)	19 ± 26	11 ± 14	12 ± 12	19 ± 26	12 ± 17	14 ± 22	-6 [-13, 1], p = 0.103
**XL-VLDL subclass concentrations** (average particle diameter of 64.0 nm)							
Lipids (μmol/L)	241 ± 168	184 ± 125	181 ± 90	212 ± 117	210 ± 152	220 ± 166	-65 [-114, -15], **p = 0.011**
Cholesterol (μmol/L)	63 ± 32	51 ± 26	51 ± 21	58 ± 24	57 ± 29	28 ± 31	-13 [-22, -1], **p = 0.005**
Cholesteryl esters (μmol/L)	36 ± 16	31 ± 13	31 ± 11	35 ± 13	34 ± 15	35 ± 15	-6 [-10, -2], **p = 0.006**
Free cholesterol (μmol/L)	27 ± 17	20 ± 13	20 ± 10	24 ± 12	23 ± 15	24 ± 16	-7 [-12, -2], **p = 0.007**
Triacylglycerol (μmol/L)	135 ± 107	100 ± 77	97 ± 54	115 ± 72	116 ± 97	121 ± 105	-39 [-71, -7], **p = 0.017**
Phospholipids (μmol/L)	44 ± 31	33 ± 23	32 ± 17	38 ± 22	37 ± 27	40 ± 30	-12 [-21, -3], **p = 0.009**
**L-VLDL subclass concentrations** (average particle diameter of 53.6 nm)							
Lipids (μmol/L)	439 ± 244	356 ± 205	353 ± 142	404 ± 184	398 ± 239	409 ± 247	-95 [-168, -22], **p = 0.011**
Cholesterol (μmol/L)	123 ± 58	101 ± 51	101 ± 38	114 ± 46	113 ± 57	115 ± 58	-24 [-41, -8], **p = 0.005**
Cholesteryl esters (μmol/L)	64 ± 27	53 ± 24	54 ± 19	60 ± 22	59 ± 27	60 ± 26	-12 [-19, -4], **p = 0.004**
Free cholesterol (μmol/L)	59 ± 32	48 ± 27	47 ± 19	54 ± 24	53 ± 31	55 ± 32	-13 [-22, -4], **p = 0.007**
Triacylglycerol (μmol/L)	233 ± 138	188 ± 115	186 ± 76	214 ± 101	211 ± 136	217 ± 141	-51 [-94, -9], **p = 0.019**
Phospholipids (μmol/L)	83 ± 51	67 ± 41	66 ± 30	75 ± 38	74 ± 48	77 ± 50	-19 [-34, -5], **p = 0.009**
**M-VLDL subclass concentrations** (average particle diameter of 44.5 nm)							
Lipids (μmol/L)	804 ± 259	706 ± 249	708 ± 192	780 ± 222	761 ± 271	777 ± 270	-109 [-184, -33], **p = 0.006**
Cholesterol (μmol/L)	249 ± 64	226 ± 56	230 ± 62	250 ± 62	241 ± 61	247 ± 65	-22 [-39, -6], **p = 0.009**
Cholesteryl esters (μmol/L)	137 ± 36	127 ± 29	130 ± 35	140 ± 35	135 ± 32	138 ± 35	-8 [-18, 1], p = 0.081
Free cholesterol (μmol/L)	112 ± 32	99 ± 30	100 ± 28	109 ± 29	107 ± 32	109 ± 33	-14 [-23, -5], **p = 0.002**
Triacylglycerol (μmol/L)	373 ± 166	320 ± 154	317 ± 102	353 ± 132	347 ± 174	353 ± 169	-63 [-113, -12], **p = 0.017**
Phospholipids (μmol/L)	181 ± 55	160 ± 51	162 ± 45	177 ± 49	173 ± 55	177 ± 56	-23 [-38, -8], **p = 0.004**
**S-VLDL subclass concentrations** (average particle diameter of 36.8 nm)							
Lipids (μmol/L)	498 ± 141	440 ± 132	442 ± 111	472 ± 118	462 ± 139	474 ± 126	-58 [-96, -19], **p = 0.004**
Cholesterol (μmol/L)	195 ± 54	174 ± 44	177 ± 46	189 ± 45	184 ± 47	189 ± 47	-18 [-31, -5], **p = 0.007**
Cholesteryl esters (μmol/L)	115 ± 34	102 ± 27	104 ± 29	110 ± 28	108 ± 29	112 ± 29	-11 [-20, -3], **p = 0.008**
Free cholesterol (μmol/L)	80 ± 20	72 ± 18	73 ± 18	78 ± 18	76 ± 19	78 ± 19	-7 [-12, -2], **p = 0.007**
Triacylglycerol (μmol/L)	177 ± 68	151 ± 66	150 ± 44	161 ± 55	158 ± 70	162 ± 59	-26 [-46, -6], **p = 0.012**
Phospholipids (μmol/L)	126 ± 33	114 ± 30	115 ± 28	123 ± 29	120 ± 32	123 ± 30	-12 [-20, -4], **p = 0.006**

[1] Values are means ± SDs; n = 28. VLDL, very low-density lipoprotein; LDL, low-density lipoprotein; HDL, high-density lipoprotein.

[2] Linear mixed-model analysis with random intercepts. Period, sex, time, treatment, and time x treatment interaction were used as fixed factors, participant as random factor, and baseline values as covariates. The interaction term was not statistically significant and, therefore, omitted from the final model. P values for the effect of treatment (mean difference [95% CI] between the mixed nut and control interventions) were reported.

Table S4. Total lipoprotein subclass concentrations following the mixed nut intervention and control period in older adults (continued)[1].

	Mixed nut intervention			Control intervention			Treatment effect[2]
	Week 0	Week 8	Week 16	Week 0	Week 8	Week 16	
**XS-VLDL subclass concentrations** (average particle diameter of 31.3 nm)							
Lipids (μmol/L)	417 ± 98	369 ± 88	368 ± 90	387 ± 90	392 ± 89	401 ± 96	-52 [-78, 26], **p < 0.001**
Cholesterol (μmol/L)	224 ± 53	119 ± 45	200 ± 50	213 ± 47	214 ± 48	219 ± 54	-26 [-39, -13], **p < 0.001**
Cholesteryl esters (μmol/L)	156 ± 37	139 ± 31	141 ± 36	150 ± 33	150 ± 34	153 ± 39	-14 [-21, -7], **p < 0.001**
Free cholesterol (μmol/L)	68 ± 16	60 ± 14	59 ± 15	63 ± 14	64 ± 15	65 ± 16	-9 [-13, -5], **p < 0.001**
Triacylglycerol (μmol/L)	74 ± 20	65 ± 20	63 ± 17	65 ± 16	67 ± 19	69 ± 17	-34 [-74, 6], **p = 0.002**
Phospholipids (μmol/L)	119 ± 30	105 ± 27	104 ± 27	109 ± 25	111 ± 26	114 ± 29	-16 [-24, -8], **p < 0.001**
**IDL subclass concentrations** (average particle diameter of 28.6 nm)							
Lipids (μmol/L)	1694 ± 321	1577 ± 284	1576 ± 326	1676 ± 318	1651 ± 295	1680 ± 340	-121 [-201, -41], **p = 0.004**
Cholesterol (μmol/L)	1194 ± 226	1112 ± 200	1109 ± 232	1184 ± 230	1165 ± 208	1185 ± 246	-86 [-146, -26], **p = 0.006**
Cholesteryl esters (μmol/L)	897 ± 172	834 ± 154	830 ± 175	888 ± 173	875 ± 159	888 ± 188	-65 [-113, -18], **p = 0.008**
Free cholesterol (μmol/L)	297 ± 56	278 ± 47	279 ± 58	295 ± 58	291 ± 50	296 ± 58	-20 [-34, -7], **p = 0.004**
Triacylglycerol (μmol/L)	112 ± 27	102 ± 26	100 ± 23	101 ± 20	104 ± 25	107 ± 23	-12 [-20, -5], **p = 0.001**
Phospholipids (μmol/L)	388 ± 75	364 ± 65	368 ± 77	387 ± 74	381 ± 69	389 ± 76	-23 [-40, -6], **p = 0.010**
**L-LDL subclass concentrations** (average particle diameter of 25.5 nm)							
Lipids (μmol/L)	2303 ± 430	2214 ± 397	2240 ± 459	2367 ± 445	2294 ± 420	2338 ± 432	-77 [-191, 37], p = 0.181
Cholesterol (μmol/L)	1684 ± 321	1624 ± 298	1648 ± 348	1747 ± 339	1685 ± 314	1719 ± 324	-49 [-138, 40], p = 0.273
Cholesteryl esters (μmol/L)	1249 ± 242	1204 ± 228	1223 ± 261	1298 ± 254	1249 ± 239	1275 ± 245	-36 [-104, 32], p = 0.292
Free cholesterol (μmol/L)	436 ± 80	420 ± 71	425 ± 87	449 ± 85	436 ± 77	443 ± 81	-13 [-35, 9], p = 0.232
Triacylglycerol (μmol/L)	116 ± 28	108 ± 25	106 ± 23	107 ± 20	110 ± 25	112 ± 23	-10 [-17, -4], **p = 0.002**
Phospholipids (μmol/L)	503 ± 90	482 ± 80	486 ± 94	513 ± 91	499 ± 86	507 ± 91	-18 [-41, 5], p = 0.121
**M-LDL subclass concentrations** (average particle diameter of 23.0 nm)							
Lipids (μmol/L)	905 ± 202	866 ± 185	878 ± 194	931 ± 197	897 ± 210	917 ± 205	-31 [-82, 21], p = 0.242
Cholesterol (μmol/L)	628 ± 143	601 ± 132	611 ± 139	650 ± 142	624 ± 153	639 ± 149	-19 [-57, 19], p = 0.316
Cholesteryl esters (μmol/L)	446 ± 110	424 ± 102	431 ± 103	460 ± 108	442 ± 120	453 ± 116	-17 [-46, 12], p = 0.252
Free cholesterol (μmol/L)	182 ± 36	117 ± 32	180 ± 38	190 ± 37	183 ± 35	186 ± 36	-3 [-12, 7], p = 0.605
Triacylglycerol (μmol/L)	39 ± 10	36 ± 8	36 ± 8	37 ± 8	37 ± 10	38 ± 9	-4 [-6, -1], **p = 0.006**
Phospholipids (μmol/L)	238 ± 50	229 ± 45	231 ± 49	244 ± 49	236 ± 49	240 ± 49	-8 [-20, 5], p = 0.217
**S-LDL subclass concentrations** (average particle diameter of 18.7 nm)							
Lipids (μmol/L)	397 ± 81	381 ± 386	386 ± 78	405 ± 78	395 ± 81	401 ± 82	-14 [-33, 4], p = 0.133
Cholesterol (μmol/L)	256 ± 53	246 ± 47	250 ± 52	263 ± 52	255 ± 54	260 ± 55	-8 [-21, 5], p = 0.234
Cholesteryl esters (μmol/L)	181 ± 40	173 ± 35	176 ± 37	185 ± 38	179 ± 40	183 ± 41	-7 [-16, 3], p = 0.166
Free cholesterol (μmol/L)	75 ± 14	73 ± 13	74 ± 16	78 ± 15	76 ± 15	76 ± 14	-1 [-5, 3], p = 0.560
Triacylglycerol (μmol/L)	17 ± 5	15 ± 5	15 ± 4	16 ± 4	16 ± 5	16 ± 5	-2 [-3, -1], **p = 0.008**
Phospholipids (μmol/L)	125 ± 23	120 ± 20	121 ± 23	127 ± 23	125 ± 23	126 ± 24	-4 [-9, 1], p = 0.085

[1] Values are means ± SDs; n = 28. VLDL, very low-density lipoprotein; LDL, low-density lipoprotein; HDL, high-density lipoprotein.

[2] Linear mixed-model analysis with random intercepts. Period, sex, time, treatment, and time x treatment interaction were used as fixed factors, participant as random factor, and baseline values as covariates. The interaction term was not statistically significant and, therefore, omitted from the final model. P values for the effect of treatment (mean difference [95% CI] between the mixed nut and control interventions) were reported.

Table S4. Total lipoprotein subclass concentrations following the mixed nut intervention and control period in older adults (continued)[1].

	Mixed nut intervention			Control intervention			Treatment effect[2]
	**Week 0**	**Week 8**	**Week 16**	**Week 0**	**Week 8**	**Week 16**	
**XL-HDL subclass concentrations** (average particle diameter of 14.3 nm)							
Lipids (µmol/L)	180 ± 79	170 ± 68	173 ± 73	173 ± 71	179 ± 66	179 ± 73	-11 [-26, 4], p = 0.134
Cholesterol (µmol/L)	93 ± 34	90 ± 29	92 ± 32	92 ± 31	94 ± 28	94 ± 31	-3 [-10, 3], p = 0.321
Cholesteryl esters (µmol/L)	69 ± 27	67 ± 23	68 ± 25	68 ± 24	69 ± 22	69 ± 24	-2 [-7, 3], p = 0.438
Free cholesterol (µmol/L)	24 ± 8	23 ± 6	24 ± 7	24 ± 7	25 ± 7	25 ± 7	-1 [-3, 0], p = 0.121
Triacylglycerol (µmol/L)	7 ± 2	6 ± 2	6 ± 2	6 ± 2	6 ± 2	6 ± 2	-1 [-2, -1], **p < 0.001**
Phospholipids (µmol/L)	80 ± 45	74 ± 38	75 ± 41	75 ± 40	79 ± 38	78 ± 41	-7 [-15, 2], p = 0.118
**L-HDL subclass concentrations** (average particle diameter of 12.1 nm)							
Lipids (µmol/L)	781 ± 338	770 ± 287	772 ± 319	773 ± 298	772 ± 280	776 ± 315	-14 [-62, 34], p = 0.549
Cholesterol (µmol/L)	363 ± 173	364 ± 144	369 ± 162	366 ± 152	365 ± 140	366 ± 157	1 [-25, 28], p = 0.919
Cholesteryl esters (µmol/L)	279 ± 135	283 ± 112	287 ± 125	284 ± 118	282 ± 109	282 ± 121	4 [-17, 25], p = 0.674
Free cholesterol (µmol/L)	84 ± 39	82 ± 33	82 ± 37	82 ± 35	83 ± 32	84 ± 36	-3 [-9, 3], p = 0.274
Triacylglycerol (µmol/L)	27 ± 9	22 ± 11	21 ± 9	23 ± 9	23 ± 10	24 ± 10	-5 [-8, -2], **p = 0.002**
Phospholipids (µmol/L)	391 ± 161	384 ± 139	382 ± 152	384 ± 142	384 ± 135	386 ± 152	-10 [-33, 12], p = 0.366
**M-HDL subclass concentrations** (average particle diameter of 10.9 nm)							
Lipids (µmol/L)	1221 ± 248	1210 ± 237	1198 ± 235	1224 ± 220	1194 ± 234	1209 ± 251	-12 [-62, 37], p = 0.615
Cholesterol (µmol/L)	607 ± 139	613 ± 122	610 ± 129	619 ± 118	601 ± 123	608 ± 135	9 [-18, 36], p = 0.513
Cholesteryl esters (µmol/L)	494 ± 112	502 ± 97	499 ± 103	506 ± 94	491 ± 100	496 ± 109	11 [-11, 33], p = 0.323
Free cholesterol (µmol/L)	113 ± 27	111 ± 26	110 ± 26	113 ± 24	111 ± 24	112 ± 27	-2 [-7, 3], p = 0.436
Triacylglycerol (µmol/L)	50 ± 13	41 ± 17	40 ± 12	43 ± 14	43 ± 15	45 ± 13	-8 [-13, -3], **p = 0.003**
Phospholipids (µmol/L)	564 ± 109	555 ± 108	549 ± 104	562 ± 104	550 ± 105	557 ± 112	-12 [-34, 11], p = 0.300
**S-HDL subclass concentrations** (average particle diameter of 8.7 nm)							
Lipids (µmol/L)	1361 ± 183	1344 ± 182	1336 ± 153	1375 ± 161	1337 ± 176	1353 ± 173	-14 [-65, 37], p = 0.579
Cholesterol (µmol/L)	546 ± 74	549 ± 68	548 ± 64	561 ± 64	543 ± 68	549 ± 69	6 [-16, 27, p = 0.594
Cholesteryl esters (µmol/L)	403 ± 57	409 ± 50	409 ± 49	418 ± 48	403 ± 52	407 ± 53	8 [-8, 25], p = 0.307
Free cholesterol (µmol/L)	143 ± 19	140 ± 20	139 ± 18	143 ± 17	140 ± 19	142 ± 18	-3 [-8, 3], p = 0.309
Triacylglycerol (µmol/L)	52 ± 16	45 ± 15	44 ± 11	47 ± 13	47 ± 14	48 ± 12	-6 [-10, -2], **p = 0.007**
Phospholipids (µmol/L)	764 ± 104	750 ± 107	744 ± 88	767 ± 94	747 ± 102	756 ± 101	-13 [-42, 15], p = 0.351

[1] Values are means ± SDs; n = 28. VLDL, very low-density lipoprotein; LDL, low-density lipoprotein; HDL, high-density lipoprotein.

[2] Linear mixed-model analysis with random intercepts. Period, sex, time, treatment, and time x treatment interaction were used as fixed factors, participant as random factor, and baseline values as covariates. The interaction term was not statistically significant and, therefore, omitted from the final model. P values for the effect of treatment (mean difference [95% CI] between the mixed nut and control interventions) were reported.

Table S5. Relative lipid concentrations within lipoprotein subclasses following the mixed nut intervention and control period in older adults[1].

	Mixed nut intervention			Control intervention			Treatment effect[2]
	Week 0	Week 8	Week 16	Week 0	Week 8	Week 16	
**XXL-VLDL subclass concentrations** (average particle diameter > 75.0 nm)							
Cholesterol (%)	37 ± 22	39 ± 24	37 ± 23	38 ± 27	42 ± 28	37 ± 20	1 [-5, 8], p = 0.666
Cholesteryl esters (%)	22 ± 16	24 ± 20	23 ± 21	25 ± 23	27 ± 24	24 ± 17	2 [-5, 9], p = 0.559
Free cholesterol (%)	15 ± 8	15 ± 7	14 ± 5	13 ± 6	14 ± 6	14 ± 5	0 [-2, 2], p = 0.947
Triacylglycerol (%)	52 ± 20	50 ± 24	52 ± 20	52 ± 24	50 ± 23	52 ± 18	-2 [-9, 5], p = 0.642
Phospholipids (%)	11 ± 5	11 ± 7	11 ± 5	10 ± 6	10 ± 6	11 ± 5	0 [-3, 2], p = 0.711
**XL-VLDL subclass concentrations** (average particle diameter of 64.0 nm)							
Cholesterol (%)	28 ± 5	30 ± 5	30 ± 4	30 ± 6	30 ± 7	30 ± 5	2 [0, 4], p = 0.055
Cholesteryl esters (%)	17 ± 4	19 ± 4	18 ± 3	18 ± 5	18 ± 6	18 ± 4	2 [0, 4], **p = 0.023**
Free cholesterol (%)	11 ± 1	11 ± 1	11 ± 1	11 ± 1	12 ± 1	12 ± 1	0 [-1, 0], p = 0.860
Triacylglycerol (%)	54 ± 6	52 ± 6	53 ± 5	52 ± 24	50 ± 23	52 ± 18	-2 [-9, 5], p = 0.642
Phospholipids (%)	18 ± 2	18 ± 2	18 ± 2	18 ± 2	18 ± 1	18 ± 1	0 [-1, 0], p = 0.155
**L-VLDL subclass concentrations** (average particle diameter of 53.6 nm)							
Cholesterol (%)	29 ± 4	29 ± 3	29 ± 2	29 ± 3	29 ± 3	29 ± 3	0 [-1, 1], p = 0.659
Cholesteryl esters (%)	15 ± 3	16 ± 2	16 ± 2	16 ± 2	16 ± 3	16 ± 2	0 [-1, 1], p = 0.478
Free cholesterol (%)	14 ± 1	13 ± 1	13 ± 1	13 ± 1	13 ± 1	13 ± 1	0 [-1, 1], p = 0.705
Triacylglycerol (%)	53 ± 5	53 ± 4	53 ± 3	53 ± 4	52 ± 4	52 ± 3	0 [-1, 1], p = 0.953
Phospholipids (%)	19 ± 2	18 ± 2	18 ± 2	18 ± 2	18 ± 2	19 ± 1	0 [-1, 0], p = 0.294
**M-VLDL subclass concentrations** (average particle diameter of 44.5 nm)							
Cholesterol (%)	32 ± 6	33 ± 5	33 ± 4	33 ± 5	33 ± 6	33 ± 5	1 [-1, 3], p = 0.296
Cholesteryl esters (%)	18 ± 4	19 ± 4	19 ± 3	19 ± 4	19 ± 4	19 ± 4	1 [0, 2], p = 0.191
Free cholesterol (%)	14 ± 1	14 ± 1	14 ± 1	14 ± 1	14 ± 1	14 ± 1	0 [0, 0], p = 0.899
Triacylglycerol (%)	45 ± 7	44 ± 6	44 ± 5	45 ± 7	44 ± 7	44 ± 7	-1 [-3, 1], p = 0.346
Phospholipids (%)	23 ± 2	23 ± 1	23 ± 1	23 ± 1	23 ± 2	23 ± 2	0 [0, 1], p = 0.584
**S-VLDL subclass concentrations** (average particle diameter of 36.8 nm)							
Cholesterol (%)	39 ± 4	40 ± 4	40 ± 3	40 ± 4	40 ± 5	40 ± 4	1 [-1, 2], p = 0.233
Cholesteryl esters (%)	23 ± 3	24 ± 3	24 ± 2	23 ± 3	24 ± 3	24 ± 2	0 [-1, 1], p = 0.452
Free cholesterol (%)	16 ± 2	17 ± 2	17 ± 2	17 ± 2	17 ± 2	17 ± 2	0 [0, 1], p = 0.097
Triacylglycerol (%)	35 ± 6	34 ± 5	34 ± 5	34 ± 6	33 ± 6	34 ± 5	-1 [-3, 0], p = 0.151
Phospholipids (%)	26 ± 2	26 ± 2	26 ± 1	26 ± 2	26 ± 2	26 ± 2	1 [0, 1], p = 0.064
**XS-VLDL subclass concentrations** (average particle diameter of 31.3 nm)							
Cholesterol (%)	54 ± 3	54 ± 3	55 ± 3	55 ± 3	55 ± 3	54 ± 2	1 [0, 1], p = 0.190
Cholesteryl esters (%)	38 ± 3	38 ± 3	38 ± 3	39 ± 3	38 ± 3	38 ± 2	1 [0, 2], p = 0.099
Free cholesterol (%)	16 ± 0	16 ± 0	16 ± 0	16 ± 0	16 ± 0	16 ± 0	0 [0, 0], **p = 0.005**
Triacylglycerol (%)	18 ± 3	18 ± 3	17 ± 3	17 ± 3	17 ± 3	17 ± 2	0 [-1, 0], p = 0.433
Phospholipids (%)	29 ± 1	28 ± 1	28 ± 1	28 ± 1	28 ± 1	28 ± 1	0 [-1, 0], p = 0.322
**IDL subclass concentrations** (average particle diameter of 28.6 nm)							
Cholesterol (%)	71 ± 1	71 ± 1	70 ± 1	71 ± 1	71 ± 1	70 ± 1	0 [-1, 0], p = 0.754
Cholesteryl esters (%)	53 ± 1	53 ± 1	53 ± 1	53 ± 1	53 ± 1	53 ± 1	0 [-1, 0], p = 0.689
Free cholesterol (%)	18 ± 1	18 ± 1	18 ± 1	18 ± 1	18 ± 1	18 ± 1	0 [0, 0], p = 0.866
Triacylglycerol (%)	7 ± 1	6 ± 1	6 ± 1	6 ± 1	6 ± 1	6 ± 1	0 [-1, 0], p = 0.285
Phospholipids (%)	23 ± 1	23 ± 1	23 ± 1	23 ± 1	23 ± 1	23 ± 1	0 [0, 0], p = 0.188

[1] Values are means ± SDs; n = 28. VLDL, very low-density lipoprotein; LDL, low-density lipoprotein; HDL, high-density lipoprotein.

[2] Linear mixed-model analysis with random intercepts. Period, sex, time, treatment, and time x treatment interaction were used as fixed factors, participant as random factor, and baseline values as covariates. The interaction term was not statistically significant and, therefore, omitted from the final model. P values for the effect of treatment (mean difference [95% CI] between the mixed nut and control interventions) were reported.

Table S5. Relative lipid concentrations within lipoprotein subclasses following the mixed nut intervention and control period in older adults[1] (continued).

	Mixed nut intervention			Control intervention			Treatment effect[2]
	**Week 0**	**Week 8**	**Week 16**	**Week 0**	**Week 8**	**Week 16**	
**L-LDL subclass concentrations** (average particle diameter of 25.5 nm)							
Cholesterol (%)	73 ± 1	73 ± 1	74 ± 1	74 ± 1	73 ± 1	74 ± 1	0 [0, 0], p = 0.491
Cholesteryl esters (%)	54 ± 1	54 ± 1	55 ± 1	55 ± 1	54 ± 1	55 ± 1	0 [0, 0], p = 0.708
Free cholesterol (%)	19 ± 1	19 ± 1	19 ± 0	19 ± 1	19 ± 1	19 ± 1	0 [0, 0], p = 0.658
Triacylglycerol (%)	5 ± 1	5 ± 1	5 ± 1	5 ± 1	5 ± 1	5 ± 1	0 [0, 0], p = 0.150
Phospholipids (%)	22 ± 1	22 ± 1	22 ± 0	22 ± 1	22 ± 0	22 ± 0	0 [0, 0], p = 0.932
**M-LDL subclass concentrations** (average particle diameter of 23.0 nm)							
Cholesterol (%)	69 ± 1	69 ± 1	70 ± 1	70 ± 1	69 ± 1	70 ± 1	0 [0, 1], p = 0.260
Cholesteryl esters (%)	49 ± 2	49 ± 2	49 ± 1	49 ± 2	49 ± 2	49 ± 2	0 [-1, 0], p = 0.670
Free cholesterol (%)	20 ± 1	21 ± 1	21 ± 1	21 ± 1	21 ± 1	20 ± 1	0 [0, 1], p = 0.120
Triacylglycerol (%)	4 ± 1	4 ± 1	4 ± 1	4 ± 0	4 ± 1	4 ± 1	0 [0, 0], p = 0.062
Phospholipids (%)	26 ± 1	27 ± 1	26 ± 1	26 ± 1	27 ± 1	26 ± 1	0 [0, 0], p = 0.847
**S-LDL subclass concentrations** (average particle diameter of 18.7 nm)							
Cholesterol (%)	64 ± 1	64 ± 1	65 ± 1	65 ± 1	64 ± 1	65 ± 1	0 [0, 1], p = 0.093
Cholesteryl esters (%)	45 ± 1	45 ± 1	45 ± 1	46 ± 1	45 ± 1	46 ± 1	0 [0, 0], p = 0.891
Free cholesterol (%)	19 ± 1	19 ± 1	19 ± 1	19 ± 1	19 ± 1	19 ± 1	0 [0, 1], p = 0.051
Triacylglycerol (%)	4 ± 1	4 ± 1	4 ± 1	4 ± 0	4 ± 1	4 ± 1	0 [0, 0], **p = 0.042**
Phospholipids (%)	32 ± 1	32 ± 1	32 ± 1	31 ± 1	32 ± 1	32 ± 1	0 [0, 0], p = 0.969
**XL-HDL subclass concentrations** (average particle diameter of 14.3 nm)							
Cholesterol (%)	54 ± 5	55 ± 5	55 ± 6	56 ± 7	54 ± 6	55 ± 7	2 [0, 4], **p = 0.021**
Cholesteryl esters (%)	39 ± 3	40 ± 4	41 ± 4	41 ± 5	40 ± 4	40 ± 4	1 [0, 3], **p = 0.025**
Free cholesterol (%)	14 ± 3	15 ± 2	15 ± 4	15 ± 3	14 ± 2	15 ± 3	0 [0, 1], p = 0.198
Triacylglycerol (%)	5 ± 2	4 ± 2	4 ± 2	4 ± 2	4 ± 2	4 ± 2	0 [-1, 0], p = 0.134
Phospholipids (%)	42 ± 7	42 ± 6	41 ± 7	40 ± 9	42 ± 7	41 ± 8	-1 [-3, 0], p = 0.136
**L-HDL subclass concentrations** (average particle diameter of 12.1 nm)							
Cholesterol (%)	46 ± 3	47 ± 3	47 ± 3	47 ± 3	47 ± 2	47 ± 2	1 [0, 1], **p = 0.025**
Cholesteryl esters (%)	35 ± 3	37 ± 3	37 ± 2	36 ± 2	36 ± 2	36 ± 2	1 [0, 2], **p = 0.003**
Free cholesterol (%)	11 ± 1	11 ± 1	10 ± 1	11 ± 1	11 ± 1	11 ± 1	0 [-1, 0], **p = 0.002**
Triacylglycerol (%)	4 ± 1	3 ± 1	3 ± 1	3 ± 1	3 ± 1	3 ± 1	-1 [-1, 0], **p = 0.017**
Phospholipids (%)	51 ± 2	50 ± 2	50 ± 2	50 ± 2	50 ± 2	50 ± 2	0 [-1, 0], p = 0.213
**M-HDL subclass concentrations** (average particle diameter of 10.9 nm)							
Cholesterol (%)	50 ± 2	51 ± 2	51 ± 2	51 ± 2	50 ± 2	50 ± 2	1 [1, 2], **p < 0.001**
Cholesteryl esters (%)	40 ± 2	42 ± 2	42 ± 2	41 ± 2	41 ± 2	41 ± 2	1 [1, 2], **p < 0.001**
Free cholesterol (%)	9 ± 1	9 ± 1	9 ± 1	9 ± 1	9 ± 1	9 ± 1	0 [-1, 0], p = 0.092
Triacylglycerol (%)	4 ± 1	3 ± 1	3 ± 1	4 ± 1	4 ± 1	4 ± 1	-1 [-1, 0], **p = 0.001**
Phospholipids (%)	46 ± 1	46 ± 1	46 ± 1	46 ± 1	46 ± 1	46 ± 1	0 [-1, 0], **p < 0.001**
**S-HDL subclass concentrations** (average particle diameter of 8.7 nm)							
Cholesterol (%)	40 ± 1	41 ± 1	41 ± 1	41 ± 1	41 ± 1	41 ± 1	1 [0, 1], **p < 0.001**
Cholesteryl esters (%)	30 ± 1	31 ± 1	31 ± 1	30 ± 1	30 ± 1	30 ± 1	1 [0, 1], **p < 0.001**
Free cholesterol (%)	11 ± 1	10 ± 1	10 ± 1	10 ± 1	11 ± 1	11 ± 1	0 [0, 0], p = 0.024
Triacylglycerol (%)	4 ± 1	3 ± 1	3 ± 1	3 ± 1	4 ± 1	4 ± 1	-1 [-1, 0], **p = 0.003**
Phospholipids (%)	56 ± 1	56 ± 1	56 ± 1	56 ± 1	56 ± 1	56 ± 1	0 [-1, 0], **p < 0.001**

[1] Values are means ± SDs; n = 28. VLDL, very low-density lipoprotein; LDL, low-density lipoprotein; HDL, high-density lipoprotein.

[2] Linear mixed-model analysis with random intercepts. Period, sex, time, treatment, and time x treatment interaction were used as fixed factors, participant as random factor, and baseline values as covariates. The interaction term was not statistically significant and, therefore, omitted from the final model. P values for the effect of treatment (mean difference [95% CI] between the mixed nut and control interventions) were reported.
